# Composition of Intestinal Microbiota in Immune-Deficient Mice Kept in Three Different Housing Conditions

**DOI:** 10.1371/journal.pone.0113406

**Published:** 2014-11-17

**Authors:** Christa Thoene-Reineke, André Fischer, Christian Friese, Dana Briesemeister, Ulf B. Göbel, Thomas Kammertoens, Stefan Bereswill, Markus M. Heimesaat

**Affiliations:** 1 Forschungseinrichtung für Experimentelle Medizin, Charité - University Medicine Berlin, Berlin, Germany; 2 Department of Micro­biology and Hygiene, Charité - University Medicine Berlin, Berlin, Germany; 3 Department of Immunology, Charité - University Medicine Berlin, Berlin, Germany; University of Leicester, United Kingdom

## Abstract

**Background:**

Abundance of commensals constituting the intestinal microbiota (IM) affects the immune system and predisposes to a variety of diseases, including intestinal infections, cancer, inflammatory and metabolic disorders. Housing conditions determine the IM and can hence influence the immune system. We analyzed how both variables affect the IM of four immune-compromized mouse lines kept under different housing conditions.

**Methodology/Principal Findings:**

We investigated the IM composition in mice by quantitative 16S rRNA RT-PCR analysis of the main fecal bacterial groups (*Enterobacteriaceae*, enterococci, lactobacilli, bifidobacteria, *Bacteroides/Prevotella* (BP) spp., *Clostridium leptum* and *coccoides* groups). Mice were homozygous (HO) or heterozygous (HE) for a targeted inactivating mutation of either the IFN-γ Receptor (R), IFN-γ, Rag1 or IL-4 genes. Overall, differences in IM composition were subtle. However, in the SPF-barrier, total eubacterial loads were higher in Rag1 HE versus Rag1 HO mice as well as in IFN-γR HE versus IFN-γR HO and WT animals. Although absent in WT mice, bifidobacterial loads were higher in HO and HE IFN-γ and Rag1 as well as IL-4 HO mice. Furthermore, BP was slightly lower in HO and HE IFN-γR and IFN-γ mice as well as in IL-4 HO mice as compared to WT controls. Interestingly, IM compositions were comparable in WT mice when kept in individual ventilated cages (IVC) or open cages (OC). IFN-γ HO and HE mice, however, had higher enterobacteria and BP loads, but lacked bifidobacteria when kept in OC versus IVC, as was the case in HO and HE Rag1 mice. In addition, Rag1 HO mice harbored higher clostridial loads when housed in OC as compared to IVC. Unexpectedly, lactobacilli levels were higher in IFN-γR mice when kept in OC versus IVC.

**Conclusion/Significance:**

Housing-dependent and immune-deficiency mediated changes in intestinal microbiota composition were rather subtle but may nevertheless impact immunopathology in experimental models.

## Introduction

Increasing evidence shows that the mammalian intestinal microbiota is a key regulator of health and disease [1]. Recent research focuses on the interrelationship between the commensal intestinal microbiota and host immunity [1], [2]. Numerous recent studies highlight the role of the microbiota composition in initiating and perpetuating acute and chronic intestinal inflammation in mice and men [3]–[6]. Differences in the commensal intestinal microbiota composition could be problematic when comparing *in vivo* mouse data derived from different research institutions [7]. Intestinal colonization in mice cannot only vary between animal facilities, but also between units within the same facility, rooms within the same unit, and even between cages within the same room [8]–[11]. Moreover, factors such as age, sex, genetic background, infection status and diet can all modulate the intestinal microbiota composition [12]. Differences in hygiene and microbial colonization status might seem negligible at the first glance, but may have a significant impact on results, particularly because infection and inflammation in immune-compromised gene-deficient mice may greatly alter experimental outcome [10], [12]–[14] as well as animal welfare. In this study we analyzed commensals in mice with severe defects in adaptive immunity, which lack both mature B cells and T cells (Rag1 deficient mice). In these mice T cells and antibodies are absent and thus cannot shape the commensal intestinal flora. We also analyzed mice with more subtle defects, namely a defect in Type 1 (IFN-γ Receptor (IFN-γR) deficient and IFN-γ deficient mice) and Type 2 (IL-4 deficient mice) helper T cells. While defects in Th1 cells are thought to affect host immunity against intracellular microbes, defects in Th2 cells are considered to affect extracellular pathogens such as parasites. To quantify how the intestinal microbiota composition of these specific immune-deficient mice are influenced by different housing conditions, we collected fecal samples from mice homozygously (HO) and heterozygously (HE) deficient for IFN-γ Receptor (IFN-γR), IFN-γ, Rag1, and IL-4 and the corresponding wildtype C57BL/6 (WT) controls under different housing conditions and quantified the main intestinal bacterial groups by 16S rRNA RT-PCR.

## Materials and Methods

### Ethics Statement

All animal experiments were conducted according to the European Guidelines for animal welfare (2010/63/EU) with approval of the commission for animal experiments of the “Landesamt für Gesundheit und Soziales” (LaGeSo, Berlin; registration number G0043/09). Animal welfare was monitored twice daily by assessment of clinical conditions and weight loss of mice.

### Mice

Female mice were used for the experiments. Rag1^−/−^ mice (strain no. 002216), IFN-γ^−/−^ and IFN-γR^−/−^ mice (strains no 002287 and 003288, respectively), IL-4^−/−^ mice (strain no. 002253), all on the C57BL/6J genetic background were obtained from the Jackson Laboratories. Wildtype mice were not generated as litter mates from the respective gene-deficient mouse line breedings. All mice were introduced into the breeding unit via embryo transfer. Mice of all genotypes were bred in parallel in the same breeding room with open cages, ensuring the same conditions in terms of hygiene and environment. Breeding was performed as described in [15].

### Housing conditions and health status of mice

Mice were kept under standardized conditions (22–24°C temperature; 55%±15% humidity) on a 12 h light/12 h dark cycle in groups of 3–5 in an enriched environment (social enrichment, structured bedding, mouse house with nested material). Food and tap water were provided *ad libitum*. Mice were kept under three different conditions as described previously [15]:

- Breeding unit (specific pathogen free, SPF): A SPF breeding barrier with open cages, accessible only to animal caretakers through locks. Caretakers showered and had to change clothes and shoes. All animals were introduced into this barrier by embryo transfer. The health status of mice was monitored in accordance to the FELASA recommendations from 2002 [16]. At least 10 animals of each hygienic unit were submitted to necropsy and serological, bacteriological and parasitological routine tests over (at minimum) quarterly intervals. For testing animals were chosen directly from the breedings. For cases in which animals cannot mount a humoral immune response, immune-competent out-breeders were used. All test results confirmed the SPF status and were negative for pathogens.
- Experimental unit with individually ventilated caging (IVC): An experimental semi-barrier room with racks of micro-isolator (Type II-L) individually ventilated cages. Cages were only opened under a laminar airflow cabinet with gloves-covered appropriately disinfected hands. The health status of mice was confirmed to be SPF in accordance with FELASA recommendations 2002 [16]. At least two immune-competent “bedding sentinel animals” of each rack were submitted to necropsy and serological, bacteriological and parasitological tests were performed in 6-months intervals. All test results proved absence of pathogens.
- Experimental unit with open cages (OC): An experimental semi-barrier with open cages, accessible only with lab coat, overshoes, gloves and cap. The health status of mice was performed in accordance with FELASA recommendations 2002 [16]. At least two immune-competent “bedding sentinel animals” of each room were subjected to necropsy and serological, bacteriological and parasitological routine tests in at least 6-months intervals. Results revealed one Flagella-positive animal out of four tested. *Pasteurella* spp. positive results had been detected once four years before that were confirmed to be *Pasteurella pneumotropica*.

Mice were screened for the following pathogens to assure the health status in the three different environments: In accordance to FELASA recommendations 2002 [16] viruses (Ectromelia virus, Lymphocytic choriomeningitis virus, Minute virus of mice, Mouse adenovirus type 1 and type 2, Mouse cytomegalovirus, Mouse hepatitis virus, Mouse parvovirus, Mouse rotavirus, Pneumonia virus of mice, Reovirus type 3, Sendai virus and Theileŕs murine encephalomyelitis virus), bacteria (*Citrobacter rodentium*, *Clostridium piliforme*, *Corynebacterium kutscheri*, *Helicobacter* spp., *Mycoplasma* spp., *Pasteurella* spp. such as *Pasteurella pneumotropica*, *Pneumocystis carinii/jiroveci*, *Salmonella* spp., *Streptobacillus moniliformis*, beta-haemolytic streptococci (not group D), *Streptococcus pneumoniae*, parasites (ectoparasites and endoparasites) and pathological lesions.

### Molecular analysis of the intestinal microbiota

DNA from fecal samples was extracted as described previously [17]. Briefly, DNA extracts and plasmids were quantified using Quant-iT PicoGreen reagent (Invitrogen, UK) and adjusted to 1 ng per µL. Abundance of the main bacterial groups of murine intestinal microbiota was assessed by quantitative real time (qRT)-PCR with group-specific 16S rRNA gene primers (Tib MolBiol, Germany) as described previously [18]–[20]. The number of 16S rRNA gene copies/ng DNA of each sample was determined and frequencies of respective bacterial groups calculated proportionally to the eubacterial (V3) amplicon.

### Statistical analysis

Medians and levels of significance were determined using Mann-Whitney U-Test. Two-sided probability (p) values ≤0.05 were considered as significant.

## Results

### Intestinal microbiota composition of mice in the SPF breeding barrier

**Figure 1:**
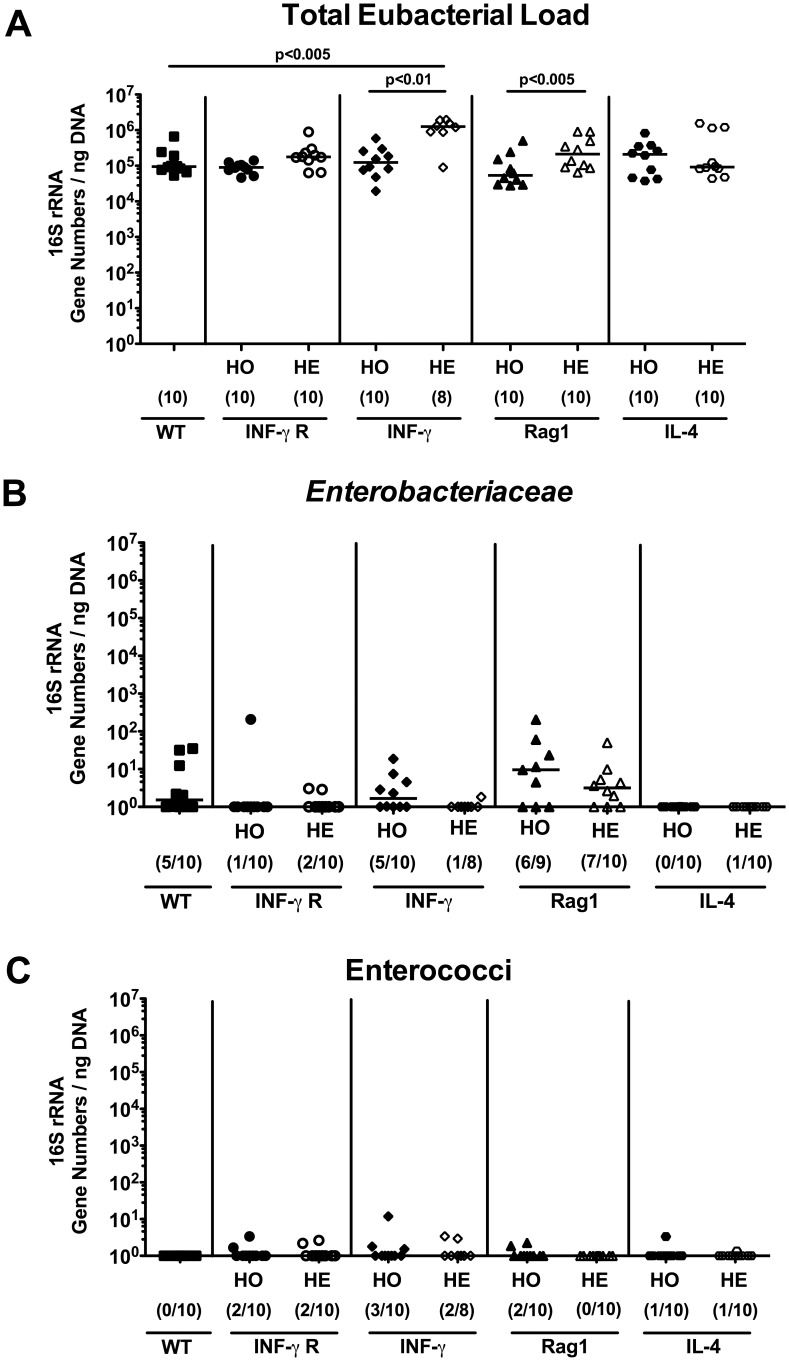
Commensal intestinal microbiota composition (total eubacterial load, *Enterobacteriaceae* and enterococci) of mice kept under SPF conditions. After twelve weeks of housing under SPF conditions, 16S rRNA of main intestinal bacterial groups was quantified by qRT-PCR in fecal samples derived from homozygous (HO; filled symbols) and heterozygous (HE; open symbols) mice deficient for IFN-γ Receptor (IFN-γ R; circles), IFN-γ (diamonds), Rag1 (triangle), or IL-4 (hexagons). Wildtype C57BL/6 (WT, black squares) served as controls. (**A**) Individual total eubacterial loads, and quantitative abundance of (**B**) *Enterobacteriaceae* and (**C**) enterococci are expressed in gene numbers per ng DNA. Medians (black bars), levels of significance (*P*-values) determined by the Mann-Whitney-U test and numbers of analyzed animals (in parentheses) are indicated.

**Figure 2:**
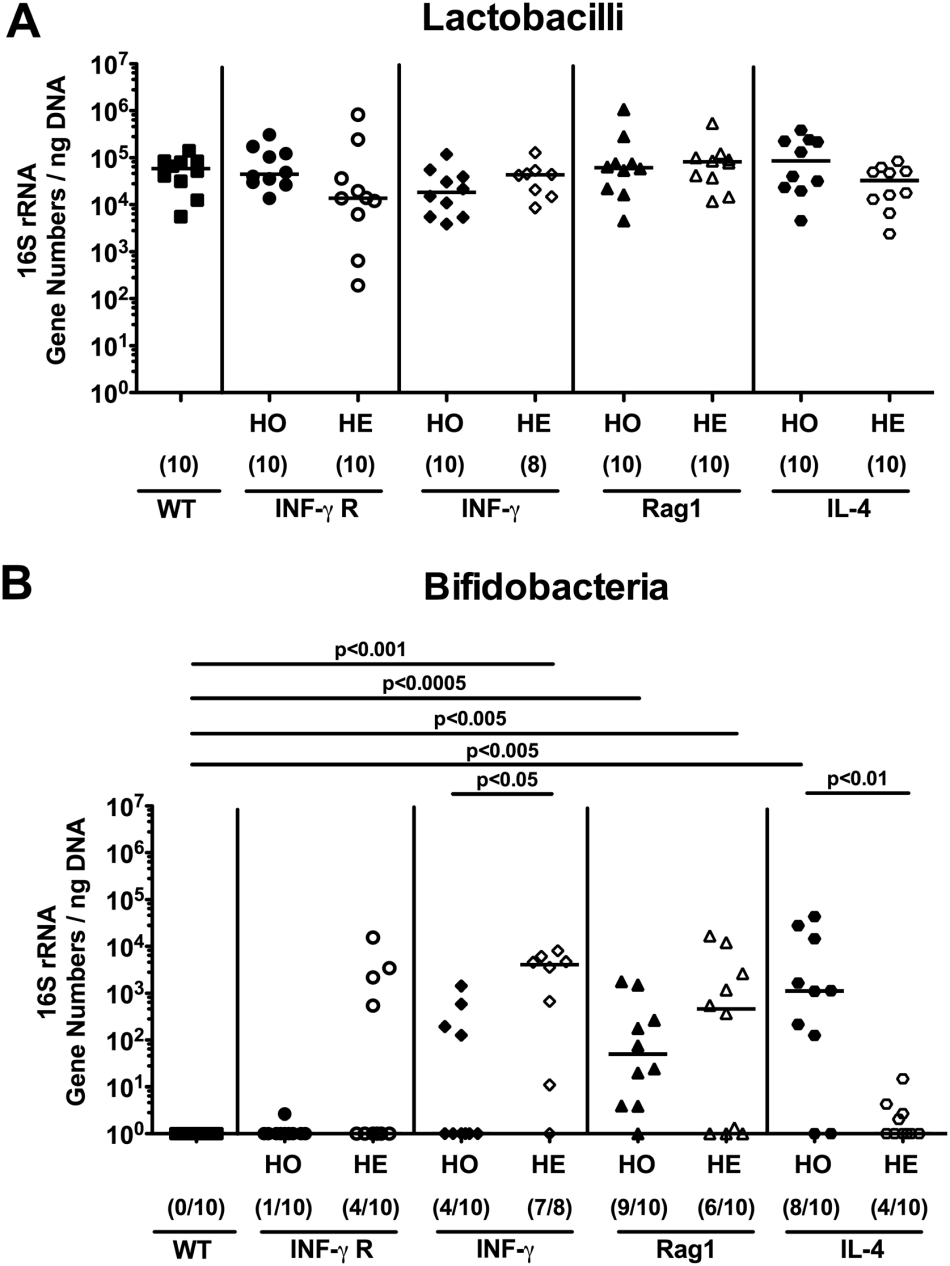
Commensal intestinal microbiota composition (lactobacilli and bifidobacteria) of mice kept under SPF conditions. After twelve weeks of housing under SPF conditions, 16S rRNA of main intestinal bacterial groups was quantified by qRT-PCR in fecal samples derived from homozygous (HO; filled symbols) and heterozygous (HE; open symbols) mice deficient for IFN-γ Receptor (IFN-γ R; circles), IFN-γ (diamonds), Rag1 (triangle), or IL-4 (hexagons). Wildtype C57BL/6 (WT, black squares) served as controls. Quantitative abundance of (**A**) lactobacilli and (**B**) bifidobacteria are expressed in gene numbers per ng DNA. Medians (black bars), levels of significance (*P*-values) determined by the Mann-Whitney-U test and numbers of analyzed animals (in parentheses) are indicated.

**Figure 3:**
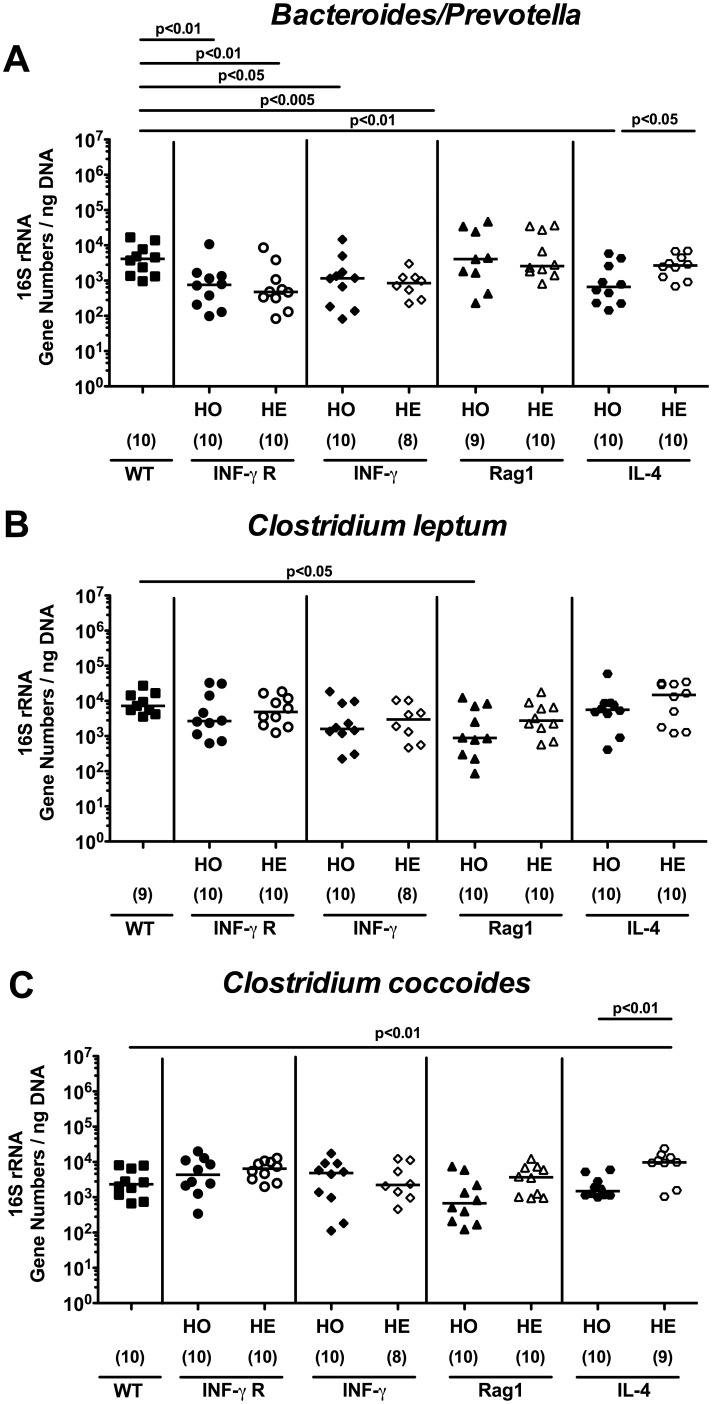
Commensal intestinal microbiota composition (*Bacteroides/Prevotella* spp., *Clostridium leptum* and *coccoides* groups) of mice kept under SPF conditions. After twelve weeks of housing under SPF conditions, 16S rRNA of main intestinal bacterial groups was quantified by qRT-PCR in fecal samples derived from homozygous (HO; filled symbols) and heterozygous (HE; open symbols) mice deficient for IFN-γ Receptor (IFN-γ R; circles), IFN-γ (diamonds), Rag1 (triangle), or IL-4 (hexagons). Wildtype C57BL/6 (WT, black squares) served as controls. Quantitative abundance of (**A**) *Bacteroides/Prevotella* spp., (**B**) *Clostridium leptum* group and (**C**) *Clostridium coccoides* group are expressed in gene numbers per ng DNA. Medians (black bars), levels of significance (*P*-values) determined by the Mann-Whitney-U test and numbers of analyzed animals (in parentheses) are indicated.

First, we investigated the commensal intestinal microbiota composition in homozygous and heterozygous mice of different genotypes kept in open cages in the breeding facility (a barrier access restricted to animal care personnel). The hygiene status of this facility is specific pathogen free according to FELASA standards 2002 [16]. Fecal samples were derived from 12-weeks-old wildtype (WT) mice, IFN-γR^+/−^ (HE) and IFN-γR^−/−^ (HO) mice, IFN-γ^+/−^ (HE) and IFN-γ^−/−^ (HO) mice, Rag1^+/−^ (HE) and Rag1^−/−^ (HO) mice, as well as IL-4^+/−^ (HE) and IL-4^−/−^ (HO) mice and subjected to 16S rRNA analysis of the most prevalent commensal intestinal bacterial groups by quantitative RT-PCR. The total eubacterial loads were slightly higher (mean difference approximately one order of magnitude) in IFN-γ^+/−^ (HE) and Rag-1^+/−^ (HE) mice as compared to the respective HO mice as well as in IFN-γ^+/−^ (HE) versus WT control animals (p<0.01, p<0.005, and p<0.005, respectively; **Fig. 1A**). However, eubacterial 16S rRNA levels did not differ between mice of the remaining genotypes. Analysis of specific bacterial groups revealed that all mice harbored comparable 16S rRNA gene levels of *Enterobacteriaceae*, enterococci, and lactobacilli (**Fig. 1B, C** **;** **Fig. 2A**). Whereas bifidobacteria were virtually absent in WT and IFN-γR^−/−^ (HO) mice, bifidobacterial 16S rRNA levels were significantly higher in IFN-γ^+/−^ (HE), Rag1^−/−^ (HO), Rag1^+/−^ (HE), and IL-4^−/−^ (HO) mice as compared to WT controls (mean differences up to 3.5 orders of magnitude; p<0.005–0.0005; **Fig. 2B**). Interestingly, bifidobacterial loads were higher in IFN-γ^+/−^ (HE) mice as compared to IFN-γ^−/−^ (HO) mice (p<0.05), but lower in IL-4^+/−^ (HE) versus IL-4^−/−^ (HO) animals (p<0.01) (**Fig. 2B**). Moreover, WT control mice displayed approximately one log higher fecal *Bacteroides/Prevotella* spp. loads as compared to IFN-γR^−/−^ (HO), IFN-γR^+/−^ (HE), IFN-γ^−/−^ (HO), IFN-γ^+/−^ (HE), and IL-4^−/−^ (HO) mice (p<0.05–0.005; **Fig. 3A**), whereas IL-4^+/−^ (HE) mice harbored one order of magnitude higher *Bacteroides/Prevotella* spp. 16S rRNA in the feces as compared to IL-4^−/−^ (HO) mice (p<0.05; **Fig. 3A**).

We next quantified the 16S rRNA levels of the fastidious obligate anaerobic Gram-positive *Clostridium* populations. Rag1^−/−^ (HO) mice exhibited slightly lower *Clostridium leptum* (cluster IV) 16S rRNA levels as compared to WT mice (p<0.05; **Fig. 3B**), whereas *Clostridium coccoides* (cluster XIV) levels were higher in IL-4^+/−^ (HE) mice as compared to IL-4^−/−^ (HO) and WT animals (p<0.01; **Fig. 3C**). However, mean differences were only approximately one order of magnitude or less.

Taken together, keeping immune-deficient mice in the same facility for 12 weeks results in only slight changes of intestinal colonization status. With the exception of bifidobacteria most changes were only approximately one order of magnitude or less.

### Effect of housing conditions (IVC versus open caging) on intestinal microbiota composition

**Figure 4:**
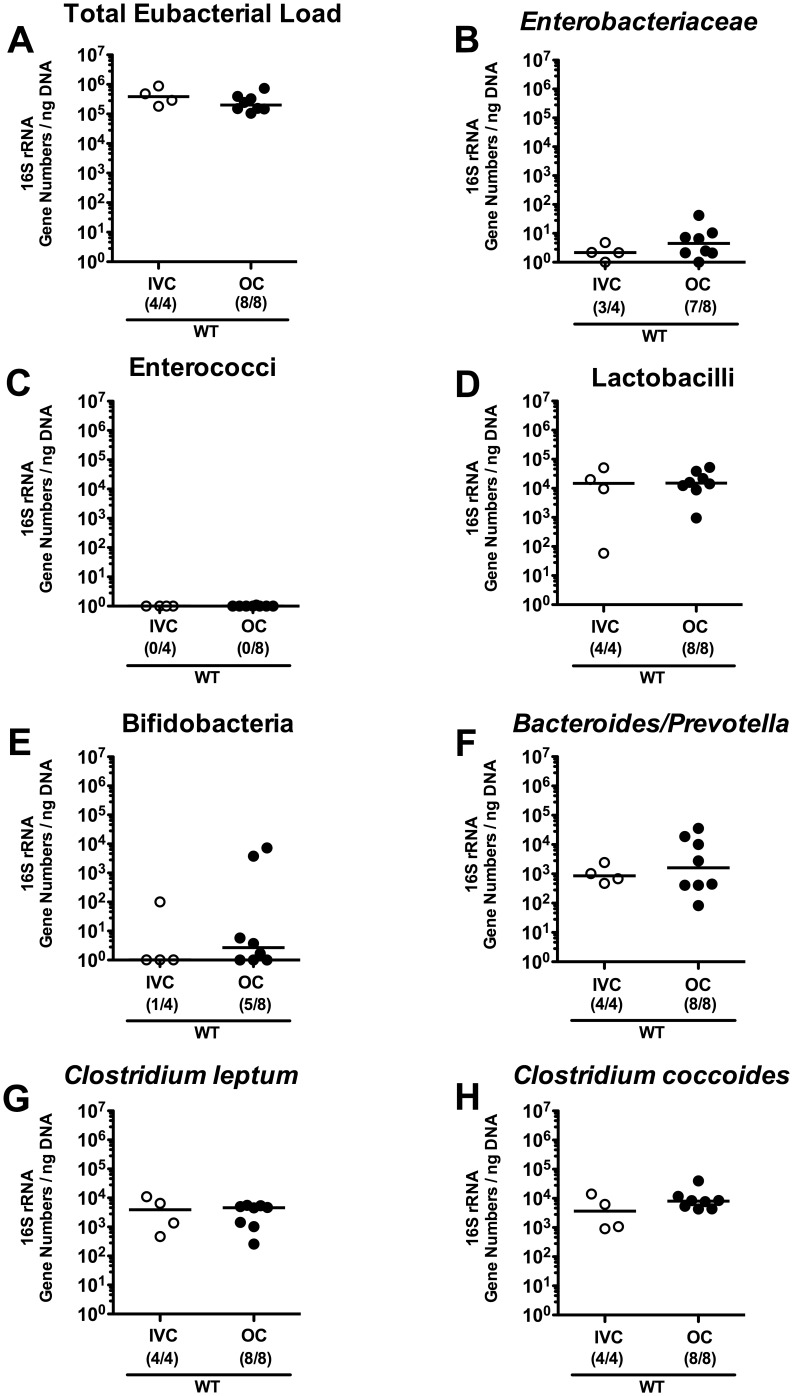
Commensal intestinal microbiota composition of wildtype mice kept in IVC or open cages. After eight weeks of housing in IVC (white circles) or open cages (OC; black circles), 16S rRNA of main intestinal bacterial groups was quantified in fecal samples derived from conventional wildtype (WT) mice. (**A**) Individual total eubacterial loads and quantitative abundance of (**B**) *Enterobacteriaceae*, (**C**) enterococci, (**D**) lactobacilli, (**E**) bifidobacteria, (**F**) *Bacteroides/Prevotella* spp., (**G**) *Clostridium leptum* group, and (**H**) *Clostridium coccoides* group are expressed in gene copy-numbers per ng DNA. Medians (black bars) and numbers of mice harboring the respective bacteria out of the total number of analyzed animals (in parentheses) are indicated.

**Figure 5:**
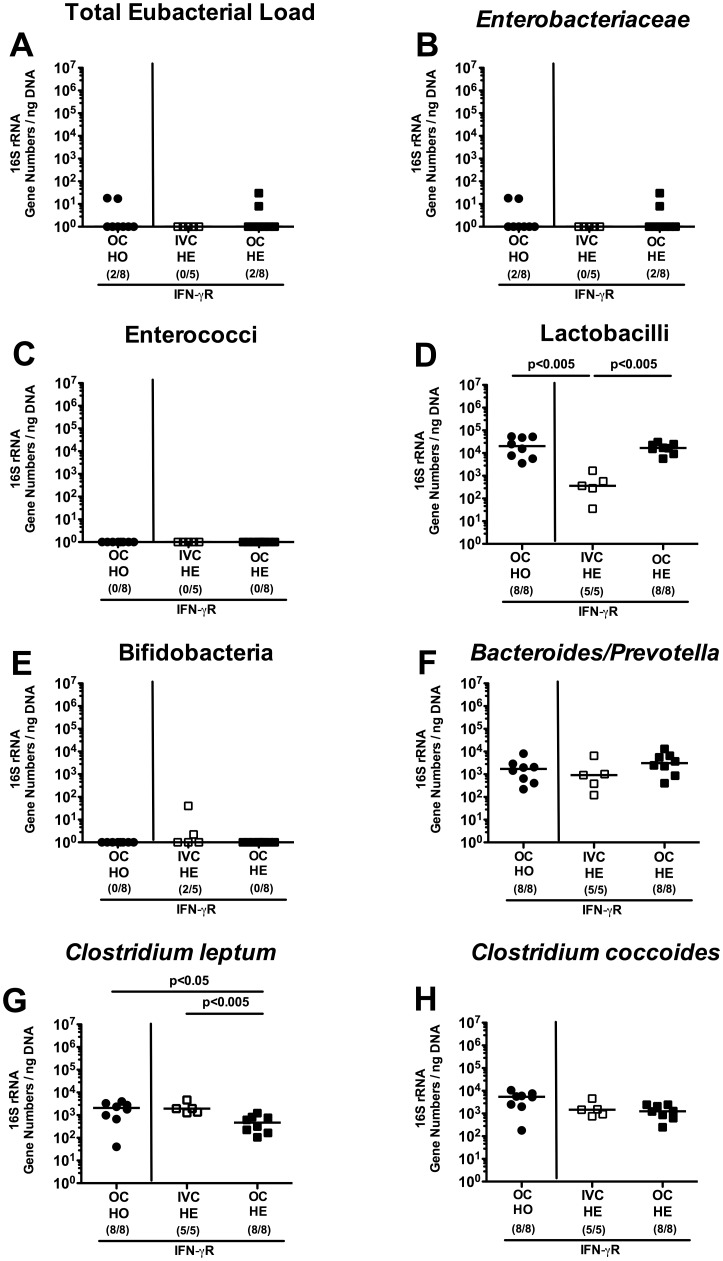
Commensal intestinal microbiota composition of IFN-γ Receptor deficient mice kept in IVC or open cages. After eight weeks housing of gene-deficient mice heterozygous (HE) or homozygous (HO) for the IFN-γ Receptor (IFN-γR) in IVC (white symbols) or open cages (OC; black symbols), 16S rRNA of main intestinal bacterial groups was quantified in individual fecal samples. (**A**) Total eubacterial loads and quantitative abundance of (**B**) *Enterobacteriaceae*, (**C**) enterococci, (**D**) lactobacilli, (**E**) bifidobacteria, (**F**) *Bacteroides/Prevotella* spp., (**G**) *Clostridium leptum* group, and (**H**) *Clostridium coccoides* group are expressed in gene numbers per ng DNA. Medians (black bars), levels of significance (*P*-values) determined by the Mann-Whitney-U test and numbers of analyzed animals (in parentheses) are indicated.

**Figure 6:**
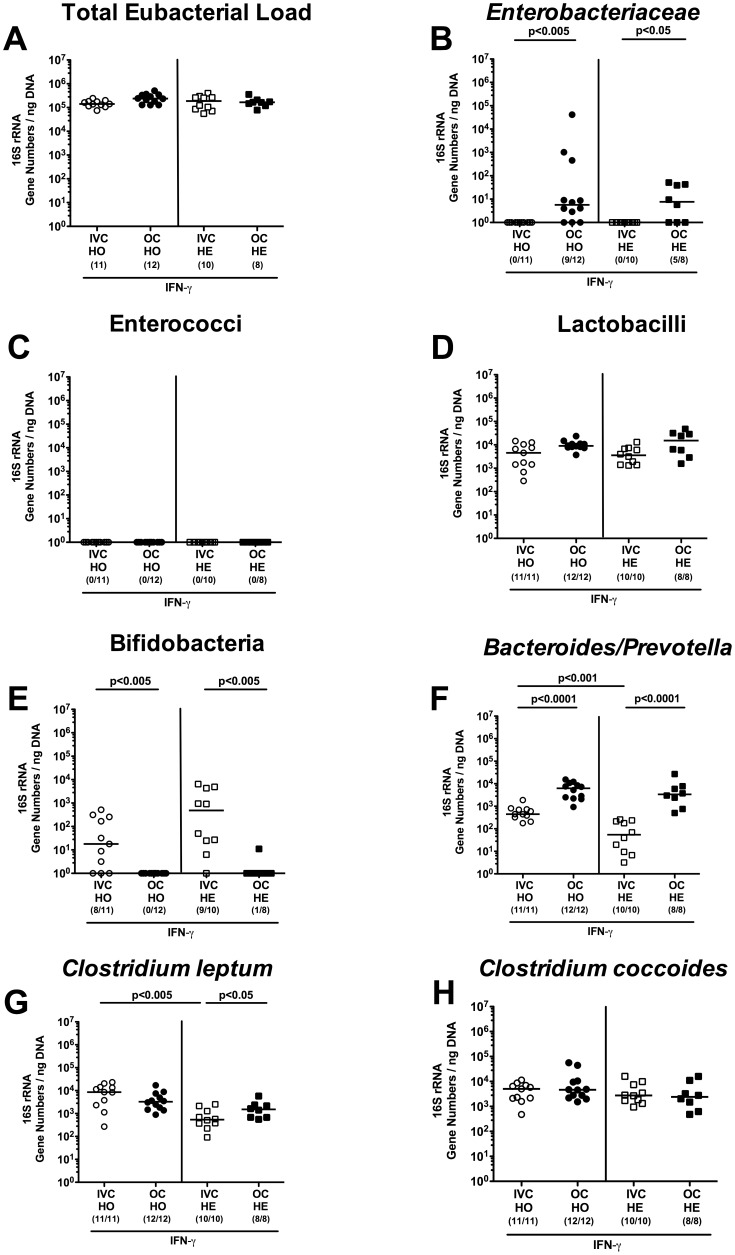
Commensal intestinal microbiota composition of IFN-γ deficient mice kept in IVC or open cages. After eight weeks housing of homozygous (HO; circles) or heterozygous (HE; squares) IFN-γ gene-deficient mice in IVC (white symbols) or open cages (OC; black symbols), 16S rRNA of main intestinal bacterial groups was quantified in individual fecal samples. (**A**) Total eubacterial loads and quantitative abundance of (**B**) *Enterobacteriaceae*, (**C**) enterococci, (**D**) lactobacilli, (**E**) bifidobacteria, (**F**) *Bacteroides/Prevotella* spp., (**G**) *Clostridium leptum* group, and (**H**) *Clostridium coccoides* group are expressed in gene numbers per ng DNA. Medians (black bars), levels of significance (*P*-values) determined by the Mann-Whitney-U test and numbers of analyzed animals (in parentheses) are indicated.

**Figure 7:**
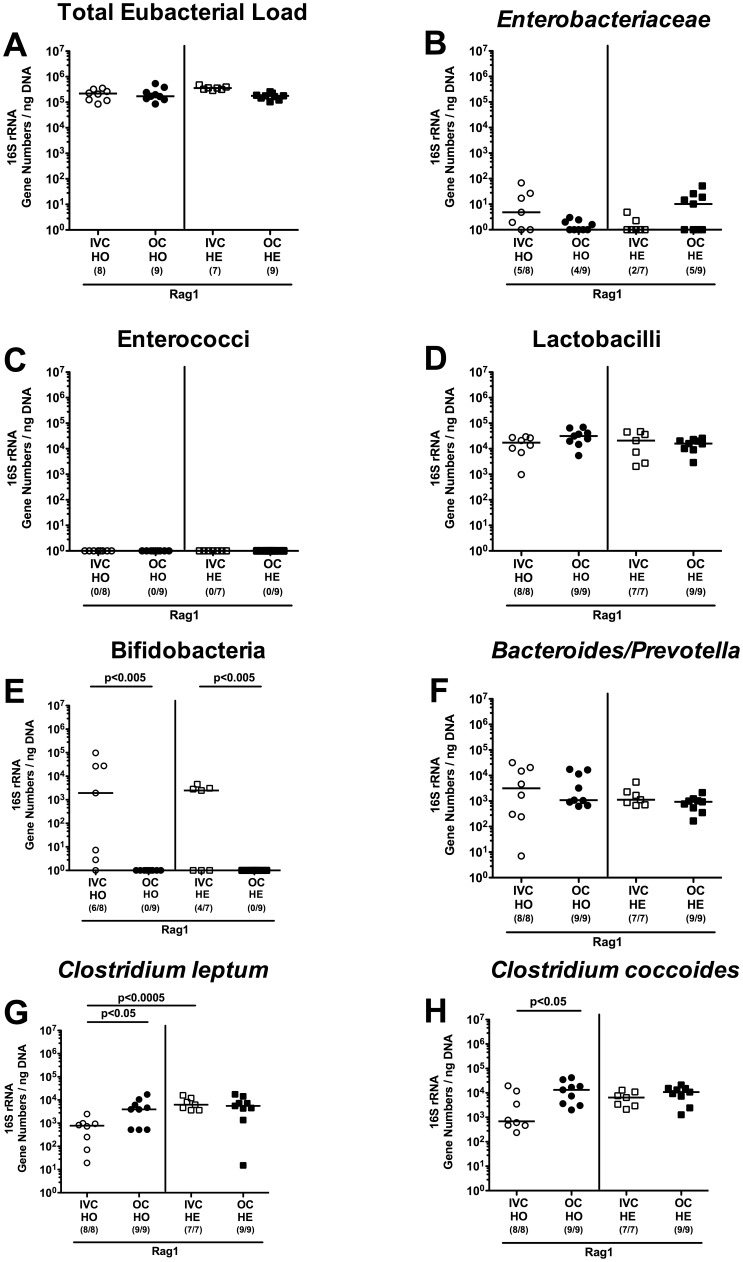
Commensal intestinal microbiota composition of Rag1 deficient mice kept in IVC or open cages. After eight weeks of housing of homozygous (HO; circles) or heterozygous (HE; squares) Rag1 gene-deficient mice in IVC (white symbols) or open cages (OC; black symbols), 16S rRNA of main intestinal bacterial groups was quantified in individual fecal samples. (**A**) Total eubacterial loads and quantitative abundance of (**B**) *Enterobacteriaceae*, (**C**) enterococci, (**D**) lactobacilli, (**E**) bifidobacteria, (**F**) *Bacteroides/Prevotella* spp., (**G**) *Clostridium leptum* group, and (**H**) *Clostridium coccoides* group are expressed in gene numbers per ng DNA. Medians (black bars), levels of significance (*P*-values) determined by the Mann-Whitney-U test and numbers of analyzed animals (in parentheses) are indicated.

We next investigated if IM composition in four different immune-deficient mouse lines varies according to housing either in open or IVC cages. To this end 4-weeks-old mice were transferred from the breeding facility into a room with open cages or into a room with IVC cages and kept there for another 8 weeks before harvesting feces samples. Remarkably, eubacterial as well as the 7 other analyzed bacterial species loads were comparable in WT control mice irrespective of housing in open or IVC cages (**Fig. 4**). In IFN-γR^+/−^ (HE) mice kept in IVC cages, lactobacilli loads were approximately 1.7 log lower when compared to IFN-γR^+/−^ (HE) and IFN-γR^−/−^ (HO) mice in open cages (p<0.005; **Fig. 5D**), whereas *Clostridium leptum* levels were approximately one order of magnitude lower in IFN-γR^+/−^ (HE) mice kept in open cages as compared to IFN-γR^+/−^ (HE) animals in IVC and IFN-γR^−/−^ (HO) mice in open cages (p<0.005 and p<0.05, respectively; **Fig. 5G**). However, 16S rRNA levels of the remaining bacterial populations were comparable (**Fig. 5**). Notably, data for intestinal colonization of IFN-γR^−/−^ (HO) mice housed in IVC cages was lacking. Interestingly, 16S rRNA levels of Gram-negative species such as *Enterobacteriaceae* and *Bacteroides/Prevotella* spp. were between one and two orders of magnitude higher in IFN-γ^−/−^ (HO) and IFN-γ^+/−^ (HE) mice housed in open as compared to IVC cages (p<0.05–0.0005 and p<0.0001, respectively; **Fig. 6B, F**), whereas bifidobacterial loads were lower for both IFN-γ^−/−^ (HO) and IFN-γ^+/−^ (HE) held in open cages than when held in IVC cages (mean differences of up to 3 orders of magnitude; p<0.005; **Fig. 6E**). Total eubacteria, enterococci, lactobacilli, and clostridial groups did not differ in IFN-γ^−/−^ (HO) and IFN-γ^+/−^ (HE) kept in either cages (**Fig. 6A, C, D, G, H**). In Rag1-deficient mice, however, housing-dependent differences in microbiota composition were even less distinct (**Fig. 7**). Bifidobacterial 16S rRNA levels were significantly lower and virtually absent in Rag1^−/−^ (HO) and Rag1^+/−^ (HE) mice kept in open cages (p<0.01; **Fig. 7E**), whereas loads of either *Clostridium* group were slightly higher (0.6–1.4 order of magnitude mean difference) in Rag1^−/−^ (HO), but not in Rag1^+/−^ (HE) mice when kept in open as compared to IVC cages (p<0.05; **Fig. 7G, H**). Furthermore, *Clostridium leptum* 16S rRNA levels were slightly higher (mean difference 0.75 orders of magnitude) in IVC housed Rag1^+/−^ (HE) as compared to IVC Rag1^−/−^ (HO) mice (p<0.05; **Fig. 7G**). The amount of the remaining bacterial groups did not differ between Rag1-deficient mice of either genotype irrespective of the housing condition.

## Discussion

It is under debate how environmental factors such as housing conditions of research animals influence the composition of their commensal intestinal microbiota. In turn, commensals are important to host immunity since they can initiate and perpetuate acute and chronic diseases, particularly in immune-compromized animals [1]–[6]. This complex issue is important for standardization of hygiene regulations to assure reproducibility and comparability of experimental settings within the same as well as between different animal facilities. In this study we performed a comprehensive quantitative molecular survey of the main intestinal commensal bacterial groups in four immune-compromized mouse lines kept in different housing conditions.

### Housing under SPF conditions

Remarkably, the overall differences in housing-dependent intestinal microbiota composition of mice were rather subtle (less than two orders of magnitude of 16S rRNA levels). Nevertheless, it is noteworthy that under SPF conditions, IFN-γ^+/−^ HE and Rag1^+/−^ HE mice displayed slightly higher total eubacterial load as compared to the corresponding homozygous knockout mice. An unexpected finding was that bifidobacteria, commonly regarded as a potentially probiotic commensal species, were clearly more abundant in IFN-γ^+/−^ HE, Rag1^−/−^ HO, Rag1^+/−^ HE, and IL-4^−/−^ HO mice as compared to WT controls. This is interesting because immune-deficiency has been typically associated with an unfavorable change in commensal microbiota. In a recent study we compared Rag1^−/−^ HO and WT mice both kept under SPF housing conditions and found that they exhibited comparable microbiota compositions [21]. Enterobacterial loads were higher in the in the earlier study [21], nevertheless the present study shows a similar trend towards higher enterobacterial 16S rRNA levels in Rag1^−/−^ HO as compared to WT mice. Therefore, we consider comparability and reproducibility sufficient and inter-assay variability acceptable. Previously, we had investigated serum levels of 10 different cytokines in the identical immune-deficient mice kept under the same three different housing conditions at our animal facility [15]. In line with this study, analysis of the intestinal microbiota revealed no major changes due to different housing conditions. Our earlier study showed that the slight imbalance in inflammatory cytokines (IL-1α, IL-5) present at 4 weeks of age (in mice with targeted mutations) normalized at 12 weeks of age. Since all fecal samples analyzed in the current study were obtained from mice that are 12 weeks of age, we can conclude that the subtle differences in the intestinal microbiota reported here were not associated with a cytokine response in these mice.

### IVC versus open cage housing

In WT mice, the fecal microbiota composition was similar under either housing condition. Remarkably, when kept in open cages (OC), bifidobacterial 16S rRNA levels were lower (and virtually absent) in IFN-γ and Rag1 deficient mice as compared to the same genotype of mice kept in IVC cages. Of note, loads of the Gram-negative bacterial groups such as *Enterobacteriaceae* and *Bacteroides/Prevotella* spp. were higher in IFN-γ deficient mice of either genotype when kept in OC versus IVC. This is important since experimental acute and chronic small and large intestinal inflammation is mediated by TLR-4 dependent signaling of commensal bacterial LPS derived from Gram-negative species [17], [18], [22], [23].

Furthermore, in OC, Rag1^−/−^ HO mice harbored 1.0–1.5 orders of magnitude higher loads of *Clostridium coccoides* and *leptum* group as compared to those in IVC housing; however these differences in clostridial populations again were rather small. The slightly higher abundance of lactobacilli (approximately 1.7 log) in IFN-γ^+/−^ HE mice kept in OC versus IVC, however, is somewhat unexpected, but comparable to the levels observed in IFN-γ^−/−^ HO mice kept in OC. Again, the differences observed in this study and in our previous analysis of serum cytokines [15] were rather subtle. Nevertheless, in the light of recent experiments implicating changes in the bacterial microbiota as an initiating or perpetuating force in many diseases, even subtle differences in commensals may influence results or change their interpretation depending on the respective experimental hypothesis.

### Importance of commensal microbiota and housing to laboratory animal health monitoring programs

FELASA has published recommendations for the health monitoring (HM) of rodent and rabbit colonies in breeding and experimental units [16], with the intention of harmonizing HM programs. These FELASA recommendations represent the highest standard for a laboratory animal HM programs and state in the preamble, that recommendations need to be optimized and adjusted periodically to meet current developments in laboratory animal medicine. Accordingly, previous recommendations have been revised and will be replaced by the new FELASA recommendation from 2014 [24]. The new recommendations focus on new definition of animal-pathogenic agents, selection of animals and tissues for testing, frequencies of sampling, commonly used test methods, interpretation of results and HM reporting. Furthermore, definition of opportunistic species, the use of sentinel animals (particularly under conditions of cage-level containment) and the interpretation and reporting of HM results are integral part of the new FELASA recommendation. So far the list of relevant pathogenic agents, testing frequencies, and literature references have been updated. Standardizing the microbial environment of experimental animals cannot only critically influence animal welfare but also the validity and reproducibility of experimental results. In this regard we consider the intestinal microbiota composition as an integral part of quality assurance.

### Outlook

In the future, more comprehensive and reliable microbiological data sets need to be obtained to unravel differences in the microbiota composition in various murine genotypes and to understand how environmental conditions can influence experimental outcomes. This will impact the environmental standardization currently practiced by FELASA so as not only to exclude pathogenic microorganisms but also to include a list of preferable commensal microbiota to improve comparability of results and assure general reproducibility of experiments. A standardized list of desired commensals may improve animal welfare and allow the respective rodent models to be more valid as well as reducing the number of animals used in the future.
